# Nearly Complete Genome Sequence of an Echovirus 30 Strain from a Cluster of Aseptic Meningitis Cases in California, September 2017

**DOI:** 10.1128/MRA.01085-19

**Published:** 2019-10-31

**Authors:** Chao-Yang Pan, Thalia Huynh, Tasha Padilla, Alice Chen, Terry Fei Fan Ng, Rachel L. Marine, Christina J. Castro, W. Allan Nix, Debra A. Wadford

**Affiliations:** a Viral and Rickettsial Disease Laboratory, California Department of Public Health, Richmond, California, USA; b Division of Viral Diseases, Centers for Disease Control and Prevention, Atlanta, Georgia, USA; DOE Joint Genome Institute

## Abstract

We report the nearly complete genome sequence of a human enterovirus, a strain of echovirus 30, obtained from a cerebrospinal fluid specimen from a teenaged patient with aseptic meningitis in September 2017.

## ANNOUNCEMENT

Echoviruses are members of the Enterovirus B species of the Enterovirus (EV) genus in the *Picornaviridae* family of nonenveloped, single-stranded, positive-sense RNA viruses. Echoviruses were named from the acronym enteric cytopathic human orphan virus at the time of their discovery in the 1950s but were later found to be associated with respiratory illness, hand-foot-and-mouth disease, and aseptic meningitis, similar to other enteroviruses (1).

According to the California Code of Regulations, meningitis cases are reportable to the California Department of Public Health (CDPH) within 1 day of identification of etiology (2). In the fall of 2017, a cluster of aseptic meningitis cases from a northern California high school were reported to the CDPH. The Viral and Rickettsial Disease Laboratory (VRDL) at the CDPH detected EV from 19 of 30 patients (63%) by real-time reverse transcription-PCR (RT-PCR), as previously described (3). We generated and analyzed partial capsid (viral protein 1 [VP1]) sequences using methods developed by Minnaar et al. (4). Fifteen of 19 (79%) EV-positive patients were confirmed to have echovirus 30 (E-30), using cerebrospinal fluid (CSF) samples. This cluster of E-30 meningitis cases is similar to previously reported E-30 aseptic meningitis cases (5, 6) in symptoms and epidemiology.

Here, we report a nearly complete genome sequence from one of the E-30-positive CSF specimens. The CSF was processed by centrifugation, 0.45-μm filtration, and nuclease treatment prior to extraction using the NucliSENS easyMAG system (bioMérieux, Durham, NC) (7). The extracted nucleic acids were then treated with DNase to yield RNA, which was subjected to random reverse transcription and PCR (7). The next-generation sequencing (NGS) library was prepared using a Nextera XT kit and sequenced on a MiSeq platform 300-cycle paired-end run (Illumina, San Diego, CA). The NGS data were analyzed using an in-house Centers for Disease Control and Prevention (CDC) pipeline which involves the removal of host sequences using bowtie2/2.3.3.1, primer removal, low-quality (below Q20) and read length (<50 nucleotides) filtering using cutadapt 1.18, read duplication removal using a Dedup.py script, *de novo* assembly using SPAdes 3.7 default parameters, and BLAST search of the resultant contigs (8). There were a total of 141,329 postprocessing FASTQ reads. The final consensus genome was inspected and annotated using Geneious v10.0.9 (9). The contig was built from 15,712 reads, assembled to an E-30 reference genome (GenBank accession number JX976773), and deemed nearly complete by comparison to the reference, and the termini were determined as part of the protocol (7). The total GC content is 48.3% for 7,155 bases. The average read coverage was 260-fold for the E-30 genome.

The genome sequence was designated E-30 USA/2017/CA-RGDS-1005. Its VP1 sequence was confirmed by the CDC Picornavirus Laboratory to be nearly identical to those of E-30s identified in an aseptic meningitis outbreak that occurred in the fall of 2017 in Nevada; it also has greater than 99% nucleotide identity to the VP1 sequences of E-30 strains from the southern United States identified by the CDC in May 2017 (GenBank accession numbers MG584831 and MG584832), as measured using the online version of blastn (https://blast.ncbi.nlm.nih.gov/Blast.cgi). The genome sequence of E-30 USA/2017/CA-RGDS-1005 shares less than 89% nucleotide identity (NI) and less than 98% amino acid identity (AI) with other publicly available E-30 sequences. The sequence contains the complete protein-coding region, with short sections in the untranslated regions (UTRs) missing due to a lack of read coverage (approximately 182 and 90 nucleotides of the 5′ and 3′ UTRs, respectively). The enterovirus polyprotein can be divided into one structural (P1-capsid) and two nonstructural (P2 and P3) regions. The polyprotein regions of the E-30 genome reported here share 96%, 88%, and 84% NI (P1, P2, and P3, respectively) with other E-30 sequences in GenBank.

### Data availability.

The E-30 sequence of USA/2017/CA-RGDS-1005 has been deposited in GenBank under the accession number MK238483. The quality-filtered FASTQ reads have been deposited in the Sequence Read Archive with the run accession number SRR10082176.

## References

[1] Abedi GR, Watson JT, Pham H, Nix WA, Oberste MS, Gerber SI 2015 Enterovirus and human parechovirus surveillance–United States, 2009–2013. MMWR Morb Mortal Wkly Rep 64:940–943. doi:10.15585/mmwr.mm6434a3. 26334674

[2] California Department of Public Health. 2019 Title 17, California Code of Regulations (CCR) §2500, §2593, §2641.5-2643.20, and §2800-2812 reportable diseases and conditions. California Department of Public Health, Sacramento, CA https://www.cdph.ca.gov/Programs/CID/DCDC/CDPH%20Document%20Library/ReportableDiseases.pdf.

[3] Shahkarami M, Yen C, Glaser C, Xia D, Watt J, Wadford DA 2015 Laboratory testing for Middle East respiratory syndrome coronavirus, California, USA, 2013–2014. Emerg Infect Dis 21:1664–1666. doi:10.3201/eid2109.150476. 26291839 PMC4550170

[4] Minnaar RP, Koen G, de Haan K, Wolthers KC, Benschop K 2012 Evaluation of two (semi-)nested VP1 based-PCRs for typing enteroviruses directly from cerebral spinal fluid samples. J Virol Methods 185:228–233. doi:10.1016/j.jviromet.2012.07.009. 22796036

[5] Croker C, Civen R, Keough K, Ngo V, Marutani A, Schwartz B, Centers for Disease Control and Prevention (CDC) 2015 Aseptic meningitis outbreaks associated with echovirus 30 among high school football players–Los Angeles County, California 2014. MMWR Morb Mortal Wkly Rep 63:1228. 25551596

[6] Holmes CW, Koo SS, Osman H, Wilson S, Xerry J, Gallimore CI, Allen DJ, Tang JW 2016 Predominance of enterovirus B and echovirus 30 as cause of viral meningitis in a UK population. J Clin Virol 81:90–93. doi:10.1016/j.jcv.2016.06.007. 27367546

[7] Ng TF, Marine R, Wang C, Simmonds P, Kapusinszky B, Bodhidatta L, Oderinde BS, Wommack KE, Delwart E 2012 High variety of known and new RNA and DNA viruses of diverse origins in untreated sewage. J Virol 86:12161–12175. doi:10.1128/JVI.00869-12. 22933275 PMC3486453

[8] Montmayeur AM, Ng TF, Schmidt A, Zhao K, Magana L, Iber J, Castro CJ, Chen Q, Henderson E, Ramos E, Shaw J, Tatusov RL, Dybdahl-Sissoko N, Endegue-Zanga MC, Adeniji JA, Oberste MS, Burns CC 2017 High-throughput next-generation sequencing of polioviruses. J Clin Microbiol 55:606–615. doi:10.1128/JCM.02121-16. 27927929 PMC5277531

[9] Kearse M, Moir R, Wilson A, Stones-Havas S, Cheung M, Sturrock S, Buxton S, Cooper A, Markowitz S, Duran C, Thierer T, Ashton B, Meintjes P, Drummond A 2012 Geneious Basic: an integrated and extendable desktop software platform for the organization and analysis of sequence data. Bioinformatics 28:1647–1649. doi:10.1093/bioinformatics/bts199. 22543367 PMC3371832

